# The andes of bladder stones: Gigantic bladder calculi in a patient with bladder outlet obstruction and hyperparathyroidism

**DOI:** 10.1016/j.eucr.2023.102416

**Published:** 2023-05-03

**Authors:** Halimat A. Olaoluwa, Hari Polenakovik, Jonathan Hakim

**Affiliations:** a Wright State University Boonshoft School of Medicine, Dayton, OH, USA; b Department of Infectious Disease, Dayton VA Medical Center, Dayton, OH, USA; c Department of Urology, Dayton VA Medical Center, Dayton, OH, USA

**Keywords:** Bladder stones, Bladder outlet obstruction, Primary hyperparathyroidism, Case report

## Abstract

60-year-old man with known chronic urinary retention (CUR) managed with clean intermittent catheterization (CIC); He presented with difficulty with CIC. A KUB revealed 13 cm in conglomeration bladder stones. Routine preoperative bloodwork revealed calcium >12 and a subsequent PTH was also elevated. Osteoporosis was confirmed on DEXA. Sestamibi parathyroid scan had increased uptake within one parathyroid gland. The patient underwent open suprapubic 92 gm prostatectomy with evacuation of 254 gm calcium phosphate bladder stones; this was followed by removal of the offending parathyroid gland. On follow up, the patient was voiding well with normalization of other symptoms related to hyperparathyroidism.

## 1 Introduction

Primary hyperparathyroidism (PHPT), an endocrinopathy, and bladder outlet obstruction (BOO), an anatomic obstruction, are two distinct conditions that can cause urinary tract stone disease. We present a case wherein 13 cm of bladder stones accumulated in an accelerated interval due to the cumulative effects of both. No other similar cases have been described in the literature.

## 2 Case presentation

**Fig. 1:**
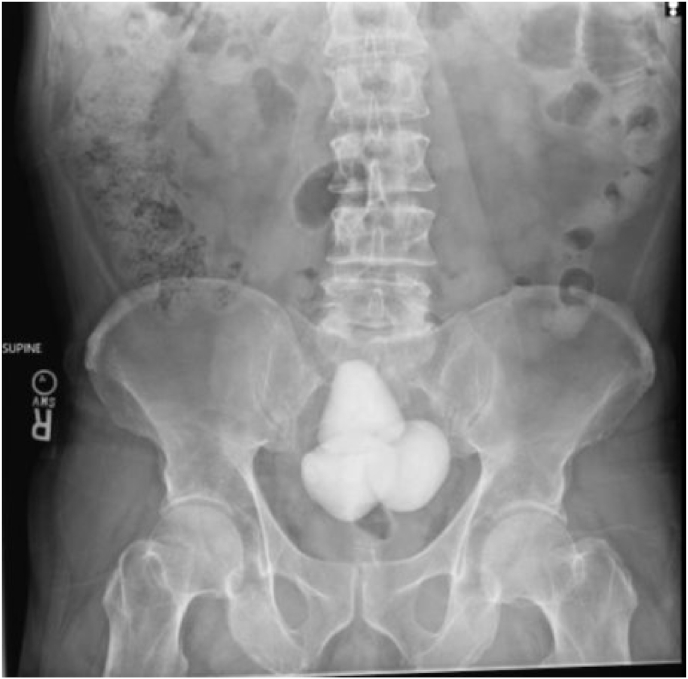
KUB on presentation.

**Fig. 2:**
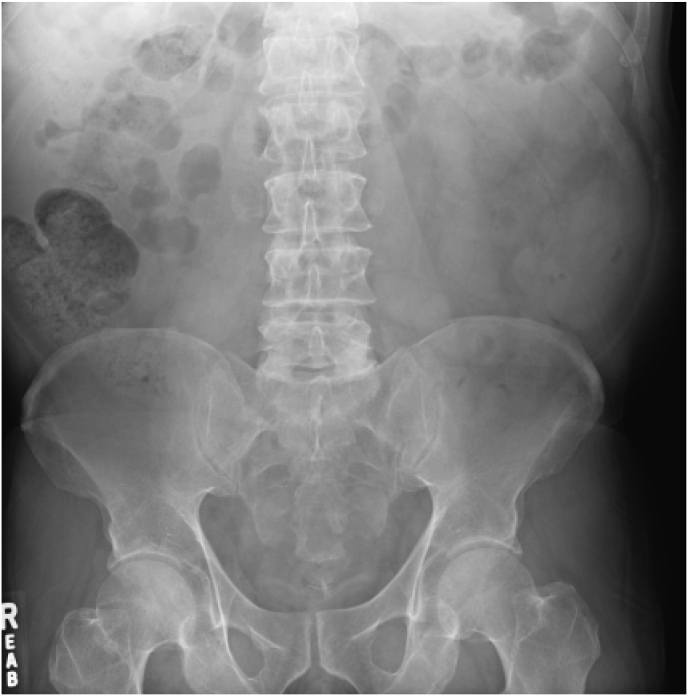
KUB 32 months prior.

The patient is a 60-year-old man with chronic urinary retention secondary to BOO, managed (CIC). He noted difficulty performing CIC due to pain. A KUB x-ray revealed 13 cm in conglomeration radiopaque bladder stones (Fig. 1). This was confirmed on direct visualization via cystoscopy. On review of imaging, the impressive bladder stones were conspicuously absent on KUB 32 months prior (Fig. 2). Previously, a transrectal ultrasonography and prostate needle core biopsy (TRUS/PNB) was performed for a chronically elevated PSA of 10 ng/mL (0–4 ng/mL). This revealed an impressive 150–200 mL prostate with benign pathology.

Routine preoperative bloodwork showed a calcium of 13.4 mg/dL (8.5–10.4 mg/dL) and a subsequent PTH was noted to be 166 pg/mL (18.4–80 pg/mL). The patient exhibited other manifestations of PHPT including chronic depression, constipation, and abdominal pain. Sestamibi parathyroid scan demonstrated increased uptake within the right inferior parathyroid gland. Osteoporosis in bilateral femoral neck was also confirmed on dual energy x-ray absorptiometry (DEXA) with lowest t-score of −3.0.

The patient underwent open suprapubic 92 gm prostatectomy with evacuation of 254 gm calcium phosphate bladder stones. This was followed by the removal of the offending benign parathyroid adenoma.

**Fig. 3:**
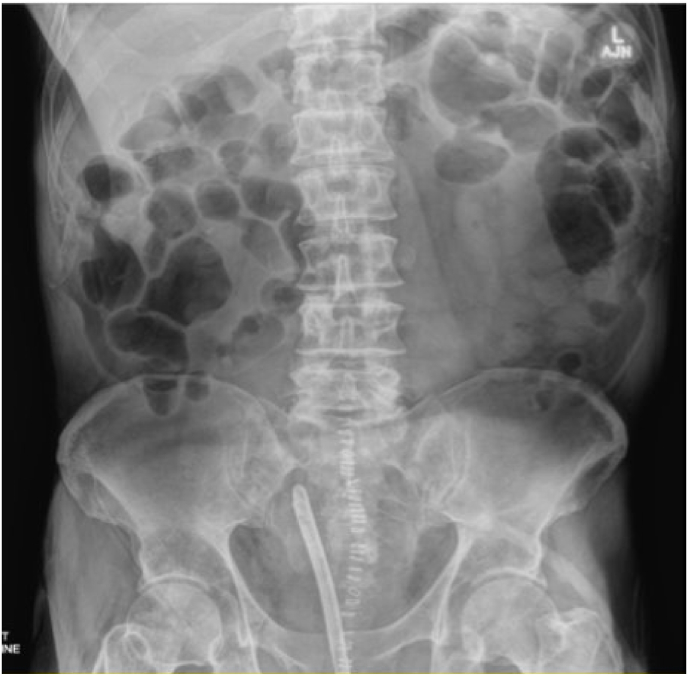
KUB post-operatively.

On follow up, the patient was voiding well with complete removal of bladder stones on KUB (Fig. 3) and had a normal calcium level of 9.9 mg/dL with PTH 52.2 pg/mL. All other clinical symptoms related to PHPT resolved post operatively.

## 3 Discussion

Urinary bladder stones represent approximately 5% of urinary tract stone disease and are mostly caused by conditions that lead to urinary stasis, including BOO and neurogenic bladder. Bladder stones tend to have a predilection towards men with peak incidence at 60 years old and can be composed of chemicals such as uric acid, calcium oxalate, calcium phosphate, and magnesium ammonium phosphate.^1^ Our patient's risk factors for bladder stones included his age (60 years old), male sex, and prostate volume.

Patients with bladder stones may present with symptoms related to BOO. This includes urinary urgency and frequency, hematuria at the end of urination, suprapubic pain, urinary retention, and sudden cessation of the urine stream.^1^ Our patient presented with pain while attempting to perform CIC. Diagnosis can be made using ultrasound, KUB x-ray, CT scan, or cystoscopy.^1^ Prostate evaluation is also recommended for men. The patient was assessed with KUB and cystoscopy which demonstrated 13 cm in conglomeration bladder stones. The prostate evaluation included a 150–200 mL prostate at the time of his negative TRUS/PNB for a chronically elevated PSA.

Our patient's blood work revealed elevated calcium and PTH raising suspicion for PHPT. PHPT is caused by a parathyroid adenoma that autonomously secretes excess PTH, leading to increased serum calcium levels and subsequent excretion of calcium in the urine. Most clinical PHPT symptoms are demonstrated in the classic “stones, bones, groans, thrones, psychiatric overtones” pattern with urinary tract stone disease, bone pain, abdominal pain, constipation, and psychiatric presentation. The urinary tract stones result from increased urinary excretion of excess calcium.^2^ Medical literature on the composition of stones formed in patients with PHPT is controversial—some authors claim a calcium oxalate predominance while others indicate a calcium phosphate predominance.^3^^,^^4^ Alkaline urine favors the precipitation of calcium phosphate stones.^4^ Our patient's bladder stones were consistent with calcium phosphate composition, and his urine pH was persistently alkaline at 6.5 and 7.0.

Up to 5% of patients with calcium stones also have PHPT. The American Urological Association (AUA) guidelines recommend screening electrolytes including calcium for patients who present with kidney stones.^5^ In addition, AUA guidelines also suggest obtaining intact PTH if suspicion for PHPT remains high, such as in the setting of elevated calcium levels and calculus predominantly composed of calcium phosphate.^5^ On careful chart review, our patient's previous stone composition was mixed calcium oxalate and phosphate from a ureteroscopy; however, he was lost follow up. Further workup including a nuclear parathyroid scan confirmed the presence of a parathyroid adenoma. DEXA scan revealed osteoporosis with a low T-score which is significant considering this is an uncommon finding in men less than 65 years old.

The preferred treatment of small volume bladder stones is the transurethral minimally invasive approach via endoscopy.^1^ However, a suprapubic approach addresses the high-volume bladder stones and the large obstructing prostate, which was the culprit of the chronic urinary retention and contributed to the bladder stone formation. An open approach was utilized in this patient because the advantage of laparoscopic robotic approach would have been nullified by the large homogenous volume of bladder stones. The urology portion was managed before the endocrine surgery out of concern for looming acute renal failure secondary to hydronephrosis. Management of any underlying conditions is also warranted. The curative treatment option for patients with PHPT is surgery with parathyroidectomy.^2^ Our patient underwent parathyroidectomy with normalization of his calcium and PTH.

Rapid accumulation of bladder stones over a 30-month period in the setting of both BOO and PHPT is rare and has not been reported in the literature, and all the patient's symptoms resolved with surgery.

## 4 Conclusion

Large bladder stone accrual in patients with anatomical and metabolic derangements is rare and needs to be addressed surgically. This case highlights and emphasizes the role of a multidisciplinary approach to tackling these complex problems.

## Funding

This research did not receive any specific grant from funding agencies in the public, commercial, or not-for-profit sectors.

## Author contribution

Halimat Olaoluwa: Writing - Original Draft, Visualization. Jonathan Hakim: Conceptualization, Writing - Review & Editing, Supervision. Hari Polenakovik: Writing - Review & Editing.

## Declaration of competing interest

None.
